# The Long Fox Memorial Lecture: The Relationship of Erythema Nodosum to Tuberculosis

**Published:** 1928

**Authors:** J. Odery Symes

**Affiliations:** Consulting Physician to the Bristol General Hospital


// The Bristol
Medico-Chirurgical Journal

"*Scire est nescire, nisi id me
Scire alius sciret.*"

WINTER, 1928.

### THE LONG FOX MEMORIAL LECTURE:

DELIVERED IN THE PHYSIOLOGICAL LECTURE THEATRE OF THE UNIVERSITY ON OCTOBER 30TH, 1928.

THOMAS LOVEDAY, LL.D., *Vice-Chancellor of the University of Bristol, in the Chair.*

BY

J. ODERY SYMES, M.D.,
*Consulting Physician to the Bristol General Hospital,*

ON

### THE RELATIONSHIP OF ERYTHEMA NODOSUM TO TUBERCULOSIS.

I VERY much appreciate the honour that has been done me in asking me to deliver the Long Fox Memorial Lecture. My subject is one that would have interested the distinguished physician in whose memory this annual lecture was founded, for he was chairman of the local branch of the National Association for the Prevention of Consumption, and one of the original founders of what is now Winsley Sanatorium. Amongst his many contributions to medical literature were articles on acute tubercle, on tuberculous phthisis, on the temperature in phthisis and tuberculosis, and on practical difficulties in the diagnosis of acute phthisis.

My personal acquaintance with Dr. Long Fox was slight, but soon after I arrived in Bristol he asked me to dine with him, and introduced me to members of the staff of the Royal Infirmary, and this subsequently led to my appointment as bacteriologist to that institution.

Newbolt, in his book, *Clifton College Forty Years Ago*, says: "Dr. Fox was one of the *great* doctors. Wherever he went pain was cheered, the dying comforted." This is an apt description, and one we should all covet. During the five years in which I watched him driving through the streets of Clifton I was constantly struck, not only by the fine intelligence of his face, but also by its beautiful benevolence. Certainly he is the only Bristol doctor whose portrait ever appeared in the pages of *Punch*, where it was included as an illustration of "a good bedside manner."

Even in Long Fox's day the problem of the nature and causation of erythema nodosum was being discussed. His friend, Dr. Harrison, who was physician and skin specialist to the Bristol General Hospital, was made President of the Dermatological Society in 1900, and took erythema nodosum as the subject of his address, describing it as an acute specific disorder akin to the exanthemata,—like them, infectious or contagious.

Dr. Alfred Lendon, of Adelaide, whose little book entitled *Nodal Fever* has been the standard and classical work on erythema nodosum since its publication in 1905, was house-surgeon at the Bristol Royal Infirmary from 1879 to 1883, and no doubt was influenced by

Long Fox's teaching, although the latter had given up his post of physician in 1877.

I may claim, therefore, that my subject is one peculiarly suitable for the Long Fox Lecture in the University of Bristol.

Owing to mistakes in diagnosis, this disease has become confused with (1) certain skin conditions, the chief of which is *erythema multiforme*, (2) *rheumatic fever*, (3) *allergic and anaphylactic manifestations.* It is necessary, therefore, briefly to discuss some of these conditions before dealing more fully with the relationship to tuberculosis, the only disease with which erythema nodosum is closely and definitely associated.

I have discussed elsewhere* the relationship to *erythema multiforme*, and all I wish to say at this juncture is that it has seldom, in my experience, been a matter of practical importance. I am altogether opposed to the view that erythema nodosum and erythema multiforme are one and the same disease, and in my series of cases I have included none in which there could be any doubt as to the differential diagnosis.

The view that erythema nodosum is a *manifestation of acute rheumatism* is very largely due to Stephen McKenzie's[1] original contribution, and his views have been copied from one text-book to another. The aches and pains, the arthritis, the fever, and the appearance of an erythematous rash are common to both diseases, but here the similarity ends. Some years ago I tried to establish a relationship between the two diseases by making a careful physical examination and detailed anamnesis of every case of erythema nodosum.

In a series of 48 cases seen at the Bristol General Hospital up to 1907 there was the possibility of a

* *Erythema Nodosum*, 1928, Messrs. John Wright and Sons, Bristol. In this book the whole subject is dealt with fully. A review appears on page 314 of this issue.

rheumatic connection in 22 per cent., if one took into account a family or personal history of rheumatic fever, chorea, or growing pains, and regarded all cardiac bruits, of whatever nature, as due to some form of rheumatic carditis. From 1907 to 1920, 50 cases of erythema nodosum were seen in hospital and private practice, and the result was much the same; 25 per cent. gave either a family or personal history of acute rheumatism, or had a cardiac bruit whilst under observation. In the majority of these cases a note was made of the fact that the bruit was probably hæmic or due to myocardial degeneration.

If it be remembered that one of every fifteen children over 7 years of age is rheumatic, then this evidence is very unconvincing. The facts opposed to the rheumatic view may be chiefly tabulated as follows:—

| | Erythema Nodosum. | Acute Rheumatic Fever. |
|---|---|---|
| Prodromata .. .. | Common. | Rare. |
| Age incidence .. .. | Second and third decades. | 5–10 years. |
| Sex .. .. .. .. .. | Females. | Males and females about equal. |
| Seasonal incidence .. | Second and last quarters. | Third quarter. |
| Evidence of infection and epidemicity .. | Considerable. | Very little. |
| Relapse and recurrence | Rare. | Frequent. |
| Carditis, chorea and nodules .. .. .. | Not seen. | Frequent. |
| Effect of salicylates .. | Very slight. | Relief. |
| Phlyctenulæ .. .. | In about 33 per cent. | None. |

Gosse[2] has made a very complete refutation of the view that erythema nodosum is a rheumatic disease. In his series of 100 cases of erythema nodosum there was not a single one of pericarditis, endocarditis, hyperpyrexia, pneumonia or nodules. Only one had chorea. He also found that the seasonal curves did not agree, nor was the age or sex incidence the same. On the same grounds Herbert Koch[3] rejects the rheumatic theory. In his series of 48 cases not one showed evidence of endocarditis or chorea.

Landau[4] conducted an investigation at the Children's Hospital, Gothenburg, from January, 1922, to September, 1926. He summarises his results as follows: In 130 cases of erythema nodosum in children from 1 to 14 years of age, only once (0·8 per cent.) had a previous rheumatic affection occurred in one and the same patient. In 136 cases of acute rheumatism, endocarditis, or chorea the anamnesis recorded erythema nodosum in 4 cases (3 per cent.). The frequency of erythema nodosum in rheumatic affections was therefore hardly any greater than can be expected in any disease of moderate frequency in childhood. Landau also confirms the objection that the age incidence and the seasonal occurrence in the two diseases are entirely different. The rheumatic theory was founded on the very flimsiest evidence, and it is rapidly becoming discredited in this country.

The question as to whether erythema nodosum is *an allergic or anaphylactic condition* arising from one or a variety of foreign proteins acting on persons of peculiar sensitiveness must also be discussed. Allergy which gives rise to such conditions as hay fever, asthma, migraine, and urticaria differs from anaphylaxis in that it may follow the first introduction of the exciting substance; it is not necessary for the person to be sensitised first. It may be hereditary, and is difficult to produce experimentally. It most closely resembles drug idiosyncrasy. Undoubtedly many of the rashes which have been mistaken for erythema nodosum are of allergic origin, such for instance as the iodide and bromide rashes, the rashes which follow the intramuscular injection of the sera, and probably the rash of erythema multiforme.

The frequent association of erythema nodosum with the infectious diseases and with sepsis suggests an allergic origin, and the fact that erythema nodosum can be produced by the injection, and particularly the intradermic injection, of tuberculin would, if it were substantiated, point to the disease being an allergic manifestation of tuberculosis, a view which we shall see later is held by Wallgren. There are, however, many weighty arguments against the allergic theory. It would be difficult to reconcile it with a definite age, sex and seasonal incidence, and one would not expect to find a regular and orderly sequence of symptoms, or to see local outbreaks or small epidemics.

Allergic diseases are extremely apt to recur, as do hay fever, asthma and migraine. Recurrences in erythema nodosum, however, though they do occur, are exceptional. The slow onset of symptoms in erythema is very unlike the speedy response to various antigens that one sees in allergic conditions. The injection of adrenalin has no effect on erythema nodosum. Another striking difference between the two conditions is the condition of the blood. In allergy there is an eosinophilia and a leucopenia. In erythema nodosum there is a slight leucocytosis and eosinophilia is absent. Of 11 recent differential blood counts, in only one did the eosinophils reach 5 per cent. In one it was 4 per cent., in one 3·5 per cent., and in all the others 2 per cent. or under. Due consideration of all the points enumerated does not strengthen or confirm the view that erythema nodosum is a condition due to allergy or any modified form of anaphylaxis.

The view that erythema nodosum is in some way *allied to tuberculosis* is very widely held on the continent of Europe, and much literature bearing on this aspect has been published in France, Germany, Austria and Scandinavia. References to the tuberculous nature of the disease have appeared from time to time in British and American medical literature, but the theory has never firmly established itself in our medical teaching, as it has done in that of other European nations. The evidence brought forward in support of this view is chiefly clinical, but there is in addition a fair amount of experimental work bearing on the subject. It has been observed clinically that sufferers from erythema nodosum have frequently been in close and immediate contact with sufferers from tuberculosis, especially phthisis, or their history shows that there is a family history of tuberculosis or a personal history of previous tuberculous illness. It is stated that it is a common occurrence to find active tuberculous lesions in sufferers from erythema nodosum, and it is still commoner to find erythema nodosum followed by an acute and frequently fatal outbreak of tuberculosis. Pleuritic effusion is the most frequent of the sequelæ, and next come phthisis and tuberculous meningitis. Such manifestations may occur immediately after the attack of erythema nodosum, but generally there is an interval of a few weeks or months. Skiagraphy has been brought in to establish the relationship between the two conditions, the presence of opaque patches in the lungs, or of enlarged and caseous glands at the hilum, in persons suffering from erythema nodosum, being accepted as evidence of its tuberculous nature.

Interesting reports based on the clinical findings have been published by many authors. Vetlesen,[5] in a comprehensive review of the literature dealing with this subject, states that of 45 cases of erythema nodosum which he has seen at the Communal Hospital in

Christiania 26·6 per cent. were regarded as tuberculous on clinical grounds. The association with pleurisy was particularly close. Of 350 patients with pleurisy 18 had previously suffered from erythema nodosum, and of 1,317 patients suffering from various forms of tuberculosis 12 had previously had erythema nodosum. In a later communication he advises that insurance companies should not accept applicants if they have suffered from erythema nodosum within four years of the time of application, for of 46 persons who had previously suffered from that malady, and who had been passed as suitable lives, 10 subsequently developed tuberculosis. Hambro's[6] figures are even more suggestive. Of 1,000 chest cases 256 were non-tuberculous in nature and none had suffered from erythema nodosum. Of the remaining 744 tuberculous cases 62 had suffered from erythema nodosum. Pleurisy preceded the erythema nodosum in 3 cases and followed it in 27. Massini[7] of Basel states that of 29 cases of erythema nodosum 3 had definite phthisis, 6 had pleuritic effusions, 1 peritonitis, 1 miliary tubercle, 1 enlarged lymph glands, 4 pyrexia of unknown origin, 1 tuberculous bone disease, 1 Bazin's disease, and 1 had skiagraphic evidence of old tubercle of the lung. That is, 19 out of 29 were tuberculous.

The *experimental* evidence bearing on the association of erythema nodosum and tuberculosis is not so convincing. It very largely consists of the results of Von Pirquet's skin test carried out upon sufferers from erythema nodosum both during and after the attack. The tests have been carried out very stringently, being sometimes repeated three or four times if the first attempts proved negative. Many observers have been content to rely upon the ordinary tuberculin test, or have placed confidence in a local reaction arising from an intradermic injection of tuberculin. More recently several investigators have tested serum obtained by blistering over fresh erythematous nodes, or from blisters raised by intra-cutaneous injections of tuberculin, and have found in the serum substances favouring the tuberculin reaction.

Excision of the nodes and their subsequent sectioning, staining, and examining for tubercle bacilli have not as a rule been successful in their detection, but Landouzy [8] in 1913 published a case in which he claimed to have been so. This report has been extensively copied in medical literature since that date.

The inoculation of guinea-pigs with material obtained from lesions of erythema nodosum has met with but scanty success. Amongst many instances of research along these lines I may mention that of Hildebrandt, [9] who induced tuberculosis in a guinea-pig with blood obtained from an erythematous node. Such results are, however, not sufficiently numerous or exact to be convincing, and it would be easy to give a long list of failures. Ernberg sectioned nodes from 4 cases and failed to demonstrate tubercle bacilli, and materials from the nodes of 2 cases were injected into guinea-pigs without result. Vetlesen inoculated guinea-pigs with material from 5 cases of erythema nodosum, and all the results were negative.

Where systematic tests were made by Von Pirquet's method the positive results are uniformly high. Wallgren[10] obtained positive reactions in 97 per cent. of his cases. He regards the appearance of erythematous nodes as an allergic sign that the patient is now sensitised to tubercle. His work was done upon children, and he regards erythema nodosum as the exanthem of tubercle. Wallgren[11] recently published the following extraordinary incident, a school epidemic of erythema nodosum which showed its intimate association with tuberculosis :—

A child suffering from phthisis was accidentally introduced to a class of 34 girls, between the ages of 10 and 11 years, and eight weeks later 18 children developed illness confining them to bed. Of these 12 had erythema nodosum and 6 had febrile attacks lasting one to seven weeks, but no other symptoms. These children were ill between March 13th and May 20th. Between May 15th and June 4th 32 of these children were carefully examined for signs of tuberculosis. Every one of them gave a positive Von Pirquet's reaction. 31 were examined with the screen; 13 showed distinct pathological hilum shading, and 4 suspicious hilum shading. Of the 11 sufferers from erythema nodosum—1 was absent—6 showed distinct hilum changes, 1 was suspicious, and 4 showed no evidence of pulmonary change.

In a private letter addressed to me in August, 1928, Wallgren says that since the publication of his article two of the children have developed pulmonary tuberculosis and one tuberculous pleurisy.

Many explanatory theories to account for the association of these two diseases have been brought forward. Erythema nodosum has been defined as a tuberculo-bacillæmia, a tuberculo-toxæmia, and as an anaphylactic or allergic reaction caused by temporary hypersensitiveness to tuberculosis toxin.

Pollak[12] went so far as to say that erythema nodosum was a special form of subcutaneous tuberculide. Camac Wilkinson[13] says he has never seen erythema nodosum apart from tuberculosis. He would apparently classify it as one of those concealed and masked tuberculous intoxications which have recently been described by Joseph Hollos,[14] of New York, and by

Liebermeister. Others have maintained that whilst erythema nodosum is a definite and specific fever, it strongly predisposes to infection by tubercle, just in the same way as do measles and influenza. Feer[15] regarded erythema nodosum as a specific fever, but suggested that tuberculosis predisposed the patient to it, and indeed was indispensable to its development. The condition, he suggested, was one of symbiosis.

It will be seen that although there is a general consensus of opinion that the two diseases are in some way allied, there is a great diversity of views to account for this association. It is not my purpose to criticise the evidence or the theories that have been advanced, but rather to lay before you my own experience with regard to the association of erythema nodosum and tuberculosis, both from the clinical and the experimental standpoints, and then to discuss briefly the conclusions to which I have been led.

My own experience in reference to the association of erythema nodosum and tuberculosis is mainly founded on 102 cases which I have seen since 1907. Previous to that date I made no examination of the patient directed especially to the detection of tuberculosis, and some cases seen since that date I have excluded, as the notes were of a very scanty nature. The diagnoses were made entirely on clinical examination or after-history, and in no instance was reliance placed exclusively in skiagraphic findings or the results of tuberculin tests. Of the 102 cases 19 were associated with tuberculosis; of these 11 showed evidence of tuberculosis at the time of the attack and 8 developed it after convalescence, 7 within seven months of the appearance of the rash and the eighth after an interval of five years. Nineteen out of 102 cases (18·5 per cent.) may not seem a very large fraction, but the association of the two diseases has been so striking and dramatic, especially where erythema nodosum preceded tuberculosis, as to leave no doubt in my own mind that the two were intimately connected. The tuberculous conditions found were :—

| | |
|---|---|
| Acute general tuberculosis .. .. .. | 2 |
| Chronic enlargement of lymph glands .. | 4 |
| Pulmonary tuberculosis .. .. .. .. | 4 |
| Pleural effusion .. .. .. .. .. .. | 5 |
| Disease of joints and bones .. .. .. | 2 |
| Tuberculous testes .. .. .. .. .. | 1 |
| Tuberculous meningitis .. .. .. .. | 1 |

Generally speaking, the acute conditions followed the appearance of the rash, and the more chronic lesions preceded it.

The following table shows the period at which symptoms appeared after the attack of erythema nodosum :—

| | |
|---|---|
| General tuberculosis | 6 months after erythema nodosum. |
| General tuberculosis | a few weeks after erythema nodosum. |
| Pleural effusion .. | 6 months after erythema nodosum. |
| ,, ,, .. | 6 weeks ,, ,, ,, |
| ,, ,, .. | 7 months ,, ,, ,, |
| ,, ,, .. | 3 months ,, ,, ,, |
| ,, ,, .. | 5 years ,, ,, ,, |
| Meningitis .. .. | 4 months ,, ,, ,, |

A brief history of some of these cases will perhaps help to illustrate the close relationship of the two conditions.

A female child aged 2½ years developed an erythema nodosum rash on January 17th, together with lymphadenitis. The temperature fell to normal on January 22nd, and she apparently made a good recovery. On February 15th the febrile symptoms returned ; a nodule was found on the occiput, and another on the thigh. The fever continued unabated, and she died in September of generalised tuberculosis.

A nurse probationer aged 19 developed the rash of erythema nodosum on April 7th. She had a severe febrile attack lasting four weeks. There were phlyctenules in both eyes. She went home on May 6th and returned to duty three weeks later. On August 12th she was found to be febrile and delirious, and was admitted to the Bristol General Hospital under my care. She died on August 21st, and the post-mortem examination showed caseating tuberculous glands at the hilum of the lung, together with acute tuberculous meningo-encephalitis.

The history of the three following cases is very instructive and striking. The patients were nurses between 20 and 25 years of age, and the histories are so similar as to exclude any possibility of a chance association between the two diseases :—

The first nurse developed erythema nodosum in March and tuberculous pleuritic effusion in October ; the second, erythema nodosum in August and tuberculous pleurisy in March ; the third, erythema nodosum in July and pleuritic effusion in October.

Lastly, I mention the case of a girl of 15 years with a history of old rheumatic carditis. She attended the Bristol General Hospital out-patient department suffering from erythema nodosum, and, after a brief delay she was admitted under my care. The rash was then fading, but the fever never declined. She died a few days after admission, and at the post-mortem examination was found to have tuberculous mediastinal glands and a generalised tuberculous infection. All these are instances of such an intimate association of the two diseases that they cannot be regarded as purely accidental.

I am indebted to medical friends for several striking instances of the association of erythema nodosum with tuberculosis. The following was communicated to me by Dr. Marion Linton :—

In a home for feeble-minded women and young children there occurred in 1922 and 1923 eleven or twelve cases of tuberculosis. During the same period there were four cases of erythema nodosum. Three of these four cases occurred in June, 1922, and in each case the erythema nodosum preceded the tuberculosis. The first, a girl of 14 years, developed erythema nodosum in June, 1922, and died of tuberculous peritonitis in December of the same year. The second, a girl of 16 years, developed erythema nodosum in June, 1922. In November she had hæmaturia, and in the early spring of 1923 she died of generalised tuberculosis. The third developed erythema nodosum in June, 1922, and subsequently developed phlyctenular conjunctivitis, keratitis, and adenitis, for which she entered the Eye Hospital in January, 1923. The fourth case was suspected of tuberculosis, but was pronounced free and healthy on October 15th, 1923. She developed erythema nodosum four days later.

Drs. Anderson and Cooper[16] published an interesting account of three sisters who contracted erythema nodosum between September 10th and October 3rd, apparently infecting one another. Their brother at the same time also had an illness with spots on his legs, but was not seen by a medical man. Dr. Cooper, who was formerly my clinical clerk, wrote in December, 1927, in reply to my inquiry, that subsequently one sister had developed phthisis, the second phthisis and pleurisy, and the third a chest complaint, the nature of which was not definitely identified. He adds : " Since then I have seen the following cases : M. T., died of miliary tuberculosis two months after an attack of erythema nodosum, and S. C., who had tuberculous glands in the neck, followed by the appearance of erythema nodosum."

My experimental results have been scanty, and, such as they are, do little to support the theory of the tuberculous origin of erythema nodosum. With von Pirquet's reaction the proportion of positive to negative results has been as three to two. Many of my cases, however, were adults, in whom the result is notoriously uncertain, and in most cases the test was done shortly after the appearance of the rash, a period which I have since learnt often coincides with a negative phase. I have never succeeded in producing a typical reaction by the intradermic injection of tuberculin.

On two occasions I have excised nodes which, after emulsification, have been injected into guinea-pigs. In neither case did tuberculosis result. Nodes from several cases have been excised, and sectioned with a view to ascertaining whether the microscopical structure suggested that the lesion was allergic or infective in character, and whether there was anything distinctly suggestive of a tuberculous origin. This work was done by Dr. Geoffrey Hadfield, Pathologist to the Bristol General Hospital (now Professor of Pathology in the London School of Medicine for Women), to whom I am deeply indebted. From his report it is evident that the skin lesions found in erythema nodosum are not caused by the presence locally of tubercle bacilli or other micro-organisms, but that it is an acute arteriolitis in the subcutaneous fat, and that the giant cells, which are present during the later stage of the nodes, do not form part of cellular systems as in tuberculosis, the whole condition being due probably to a soluble toxin.

This finding, whilst it negatives the view that erythema nodosum is a tuberculo-bacillæmia, is not opposed to the theories of tuberculo-toxæmia, or allergic reaction due to toxins from other micro-organisms.

It will be evident that, although clinically there appears to be a close relationship between erythema nodosum and tuberculosis, this can be demonstrated in but a small proportion of the cases, various authors estimating this proportion between 10 and 20 per cent. If one takes into account the results of tuberculin tests and skiagraphy, then this percentage is considerably raised. I cannot but think, however, that to classify a patient as tuberculous simply on the evidence of von Pirquet's reaction or of skiagraphic shadows is a very bad practice, and in our present state of knowledge quite unjustifiable.

At the end of 1927 I tried to ascertain the fate of 36 cases of erythema nodosum who had been in-patients under my care at the Bristol General Hospital since 1913. Fourteen of these could not be traced, but of the remaining twenty-two, one had contracted phthisis, one had suffered from pleuritic effusion, and one had a severe and long pulmonary attack, the nature of which was not definitely decided. In my private practice I found the same state of affairs; the majority of those who have suffered from erythema nodosum are alive and well to-day, and have shown no evidence of tuberculosis, although under observation in some cases for more than twenty years.

That there is a connection between erythema nodosum and tuberculosis I am convinced, and the whole point of my lecture is to impress this fact upon the medical profession generally; but to say that erythema nodosum is a tuberculous disease, that it is the exanthem, or the first allergic sign of tuberculosis, is an exaggeration and overstatement of the case.

Unlike tuberculosis, erythema nodosum has a definite age, and a still more definite sex incidence, a period of incubation and an ordered series of symptoms. There is a marked tendency for cases to occur in the early spring months, and from time to time one sees groups of cases and small epidemic outbreaks. Recovery is generally complete and final, and the mortality is very low, quite negligible if we exclude deaths from tuberculosis. Individuals may have repeated attacks, and several members of a family may have attacks, without serious injury to health and without the subsequent appearance of tuberculosis.

The following two histories illustrate this very important point:—

Mrs. A. (whose case was communicated to me by Dr. Winifred Nott), aged 40 years, generally enjoyed good health, but had suffered two miscarriages due to antepartum hemorrhages. Her eldest child had asthma. There was no personal or family history of tuberculosis. She was seen in March, 1927, suffering from a typical attack of erythema nodosum, from which she made a complete recovery. Her history was as follows: Her first attack of erythema nodosum was at 18 years of age. It was a severe one and complicated a badly swollen arm from vaccination. She was away from work for three months. Her second attack was when 21 years of age; her third attack at 24 years; her fourth attack was slight, and she did not consult a doctor; her fifth attack when 30 years old occurred during pregnancy; the seventh attack when 37 years old occurred in the first month of pregnancy and was unusually severe. Such a history might well be quoted to support the allergic theory of causation, but it is to my mind a striking refutation of the view that this disease is a modification of tuberculosis. It is impossible to conceive that a patient should suffer eight attacks of acute tuberculosis, make a complete recovery from each, and yet exhibit, as did this patient, no signs of latent tubercle.

Or take the case of the following family, with whom I have been intimately connected professionally for thirty years.

The mother had rheumatic fever in 1883 and erythema nodosum in 1886. Of the ten children three were boys, and none of these suffered from erythema nodosum. The eldest daughter had tonsillitis in December, 1899, and this was followed by erythema nodosum, arthritis, pericarditis, and endocarditis, from which she died in March, 1900. The fourth daughter had an attack of erythema nodosum in India in 1925, and I saw her with a mild attack in 1927. The fifth daughter had erythema nodosum in 1920, and other attacks in Egypt in 1921 and 1922. The sixth daughter I saw with attacks of erythema nodosum in 1915, 1920 and 1922. The only evidence of tuberculosis in the family has been that the third daughter had suppurating glands in the neck in childhood. She has never had erythema nodosum, and there has never been any discharge from the neck wounds for more than thirty years.

A point which is strongly opposed to the tuberculous theory is that in countries where tuberculosis is common amongst the coloured natives erythema nodosum is extremely rare or comparatively unknown. I have made inquiries from several men who have practised for twenty years in India, and they tell me that erythema nodosum is rarely seen ; and in Natal, where the natives and Indians are both unusually susceptible to tuberculosis, not one of my correspondents had seen erythema nodosum in coloured patients.

I have mentioned previously that some authorities maintain that erythema nodosum is a modification of erythema multiforme, or that the two are one and the same disease. I have, however, never met with anyone who was prepared to maintain that erythema multiforme was a tuberculous disease.

It is difficult to imagine that a comparatively rare and curable condition such as erythema nodosum is identical with a disease so common and incurable as tuberculosis. Summarising the pros and cons of this matter, I conclude that the association between the two diseases is beyond dispute. This association is not invariable ; indeed, it is the exception rather than the rule. *Nevertheless, erythema nodosum must always be regarded as a danger signal, as a timely warning that tuberculosis may exist or may develop.*

This being the case, the question naturally arises as to whether there is anything distinctive or suggestive in the attack which is the precursor of tuberculosis. This cannot be definitely answered, but undoubtedly tuberculosis is more likely to follow erythema nodosum in children and young adults, and in those in whom there has been a family or personal history of tuberculosis; any long period of pyrexia before the appearance of the rash, a sustained high temperature with slow development or long continuance of the rash, especially if it be in an atypical site; these, and a prolonged convalescence, all suggest the possibility that tuberculosis may be a sequela. It has been thought that the prognosis was worse if erythema nodosum developed in a patient obviously suffering from tuberculosis, but in my own experience the onset of tuberculosis *after* the development of the nodal fever is the more acute and grave condition. While it is possible that tuberculosis prepares the soil for erythema nodosum, it seems much more likely that erythema nodosum prepares the soil for tuberculosis; or if centres of latent disease exist in the bronchial glands or elsewhere, it lights them up to renewed and often fatal activity. The danger of this tuberculous infection is greatest during the first six months following the appearance of the rash, the greater number of attacks being recorded during this period, and the tendency to acute generalisation being then most marked. No patient can be regarded as safe until three or four years have lapsed, and during that time everything must be done to build up his power of resistance.

The Long Fox Lectures were founded not only to perpetuate the memory of a great physician, but in order that new facts, and fresh additions to our knowledge might be brought before our profession, for the relief of suffering. If I have been able to-night to impress upon your minds this most subtle but most certain association between erythema nodosum and tuberculosis, then the lectures are carrying on the work that Long Fox himself performed so well, and for so many years, in our midst.

REFERENCES.

[1] *Trans. Clin. Soc.*, vol. xix.

[2] *Pract.*, 1913, xci., p. 240.

[3] *Ztschr. f. Kinderh.*, 1925–6, xl., p. 584.

[4] *Acta Paediatr.*, 1927, vi., 3–4; *Jahrb. f. Kinderheilk*, 1927, p. 117.

[5] *Tubercle*, 1922, iii., p. 433.

[6] *Acta Tuberc. Scandin.*, 1927, iii., p. 1.

[7] *Med. Woch.*, 1927, lvii., p. 708.

[8] *Presse Méd.*, 1913, xxi., p. 425.

[9] *Münch. Med. Woch.*, 1907, p. 310.

[10] *Acta Med. Scandin.*, 1926, suppl. xvi., p. 363.

[11] *Jahrb. f. Kinderheilk*, 1927, p. 313.

[12] *Wien. Klin. Woch.*, 1912, xxv., p. 1223.

[13] *Brit. Med. Jour.*, 1928, i., p. 749.

[14] *Tuberculous Intoxications*, p. 39. Edinburgh: E. S. and A. Livingstone, 1927.

[15] *Schweiz. Med. Woch.*, 1926, vii., p. 682.

[16] *Brit. Med. Jour.*, 1921, ii., p. 863.

